# N-Source Determines Barley Productivity, Nutrient Accumulation, and Grain Quality in Cyprus Rainfed Agricultural Systems

**DOI:** 10.3390/ijerph20053943

**Published:** 2023-02-23

**Authors:** Michalis Omirou, Dionysia Fasoula, Marinos Stylianou, Antonis A. Zorpas, Ioannis M. Ioannides

**Affiliations:** 1 Department of Agrobiotechnology, Agricultural Research Institute, P.O. Box 22016, Nicosia 1516, Cyprus; 2 Department of Plant Breeding, Agricultural Research Institute, P.O. Box 22016, Nicosia 1516, Cyprus; 3 Laboratory of Chemical Engineering and Engineering Sustainability, Faculty of Pure and Applied Sciences, Open University of Cyprus, Giannou Kranidioti 89, Latsia, Nicosia 2231, Cyprus

**Keywords:** barley, compost, manure, organic fertilization, principal component analysis

## Abstract

The Eastern Mediterranean and Middle East (EMME) region is already experiencing the negative effects of increased temperatures and the increase in prolonged drought periods. The use of organic fertilization could be a valuable tool to meet the main challenges of climate change and maintain the productivity, quality, and sustainability of rainfed agricultural ecosystems. In the current study, we compare the effect of manure, compost, and chemical fertilization (NH_4_NO_3_) on barley grain and straw yield in a field study for three consecutive growing seasons. The hypothesis that the barley productivity, nutrient accumulation, and grain quality remain similar among the different nutrient management strategies was tested. The results showed that both growing season and type of nutrient source significantly affected barley grain and straw yield (F*_6,96_* = 13.57, *p* < 0.01). The lowest productivity was noticed in the non-fertilized plots while chemical and organic fertilization exhibited similar grain yield, ranging from 2 to 3.4 t/ha throughout the growing seasons. For straw, the use of compost had no effect on the yield in any of the growing seasons examined. The use of manure and compost had a significant effect on grain macro- and micronutrient content but this was highly related to growing season. Principal component analysis (PCA) clearly demonstrated the discrimination of the different type of fertilization on barley performance during the course of the study, while the application of compost was highly associated with an increase in micronutrients in grain samples. Furthermore, structural equational modeling (SEM) showed that both chemical and organic fertilization had a direct positive effect on macro- (r = 0.44, *p* < 0.01) and micronutrient (r = 0.88, *p* < 0.01) content of barley grain and a positive indirect effect on barley productivity through N accumulation in grain (β = 0.15, *p* = 0.007). The current study showed that barley grain and straw yield was similar between manure and NH_4_NO_3_ treatments, while compost exhibited a residual positive effect causing an increase in grain yield during the growing season. The results highlight that N fertilization under rainfed conditions is beneficial to barley productivity through its indirect effects on N accumulation in grain and straw, while it improves grain quality through the increased accumulation of micronutrients.

## 1. Introduction

Increasing temperature and prolonged drought periods are negatively affecting soil properties and functions in semi-arid and arid regions worldwide. For example, the increase in temperature is expected to accelerate soil organic matter decomposition through enhanced microbial activity [1,2]. Drought, on the other hand, combined with intensive and heavy rainfall events destroys soil aggregates [3], increases soil erosion [4,5], enhances nutrients losses [6], and increases soil salinity [7]. Through these changes, the productivity of rain agricultural ecosystems in the Eastern Mediterranean and Middle East (EMME) region is negatively affected, driving economic and social impacts which, in turn, are enhancing food insecurity. 

Barley is one of the most important rainfed cereal crops in the EMME region and the main cereal for Cyprus’s agricultural sector, representing the 57% of the total cereal cultivated area of the country. Securing barley productivity locally is of paramount importance for the feed and the food security of the country. Recognizing that low water availability is the main limiting factor for barley ecosystems, and that climate scenarios are projecting pro-longed drought periods and higher temperatures in the EMME region, there is an urgent need to explore and re-innovate agricultural practices. 

The use of organic amendments such as composts, liquid and solid manure, as well as plant residues and green manure, could improve soil health and its ability to sustain agricultural ecosystems productivity in the long-term in semi-arid and arid regions [8]. Indeed, previous studies showed that the use of organic amendments could increase soil water retention [9,10,11,12] and improve soil aggregation [13] along with soil’s ability to infiltrate more water, thereby reducing water run-off and soil erosion. As well as the beneficial effect of organic amendments on soil physical properties that are related to water use efficiency, they are the main nutrient source and consist of a major tool for sustainable nutrient management schemes worldwide [14,15]. Numerous studies showed that the use of organic amendments has a positive effect on the performance of barley crops in arid and semi-arid environments [16,17,18,19].

Most of the studies evaluated the impact of organic amendment on barley grain or straw yield and only limited number of studies focused on the effect of organic amendments on barley grain macro- and micronutrient content [20,21]. Macro- and micronutrients grain content is an important quality indicator, whether the grain is used as animal feed or intended for human consumption. It is well established that micronutrients such as Zn, Mn, Cu, and Fe are vital components of numerous cellular processes in plants, animals, and humans [22], and there is a growing interest for increasing the content of these nutrients in feed and food products. The concentration of macro- and micronutrients in cereal grain is highly cultivar-specific, but they are also affected by environmental conditions [23] and agricultural practices [24]. For example, the addition of nitrogen had a positive effect on several microelements in wheat grain [24,25,26]. These findings suggest that nitrogen management could be an effective agronomic tool to improve grain micronutrient content in cereals [27].

The main objective of the current study was to assess how organic amendments manure and compost are affecting barley performance compared with the ammonium nitrate. Additionally, the study presents how grain macro- and micronutrients are affected from seasonality and the type of N source. Finally, the interplay between type of N source on nutrient grain content and the performance traits of grain and straw yield was investigated.

## 2. Methodology

### 2.1. Experimental Design

The current study was performed in the Experimental Station of Agricultural Research Institute at Acheleia Paphos in three consecutive growing seasons, namely between 2016 and 2018, during which “Achna”, a local barley variety, was used. In detail, the soil consisted of 22% sand, 26% silt, and 52% clay with pH (1:2.5 soil: water slurry) value of 8.4. Briefly, three different types of N source were examined based on the common farmer practices in a complete block randomized design with three replicates of 21 m^2^ plots. The N-source types used every year in the same plot were: (i) goat-composted manure (MAN) that was applied, at the rate of 6 t/ha (101 kg N/ha) 10 days before seeding, with a C/N equal to 18.7 (ii) compost (COM) derived from plant residues that was applied at the rate of 9.6 t/ha (111 kg N/ha) 10 days before seeding, with a C/N equal to 24.6 and (iii) chemical fertilization in the form of ammonium nitrate (FER) that was applied at the rate of 180 kg/ha (60 kg N/ha) as a reference, at seeding. Both organic fertilizers were manually applied in experimental plots and incorporated into the soil using rotary hoes. Control (CNT) plots did not receive any external nitrogen input to evaluate the response of barley to the different N-source management practices. Climatic data of monthly rainfall, mean minimum, and maximum temperatures were recorded from the meteorological station of the Experimental Station of ARI at Acheleia (Supplementary Figure S1). Barley was seeded late November–early December every growing season and harvested in June next year.

### 2.2. Plant and Soil Analysis

Barley straw and grain samples were collected during the three growing seasons from each plot after harvest. Samples were dried at 70 °C until constant weight was achieved, and subsequently ground. Total N was determined following the Kjeldahl method in both grain and straw samples. The determination of Ca, Mg, Mn, Cu, Zn, and Fe was performed only for barley grain samples, using atomic absorption spectrometry (AAS) after sample digestion with 65% HNO_3_ using microwave digestion process. Five topsoil (0–10 cm) samples from each experimental plot were collected and pooled forming one composite sample before the application of any N-source every growing season. Samples were transferred in the lab, sieved at 2 mm, and analyzed for total N following the Kjeldahl method, the available K, and P-Olsen [28].

### 2.3. Statistical Analysis

Two-way analysis of variance (ANOVA) was employed to test the main and interactive effects of growing season and N-source type on grain and straw yield, total N as well as on the macro- (Ca, Mg) and micronutrients (Cu, Mn, Fe and Zn) determined. Post hoc analysis was implemented using Tukey HSD criterion at a = 0.05. Two-way ANOVA and descriptive statistics were performed using R packages *dplyr, tidyverse,* and *rstatix.*

Principle component analysis (PCA) was also performed to reveal any clustering between the treatments within the growing seasons and evaluate any relationship between macro- and micronutrients with grain and straw yield using R packages *FactoExtra* and *FactoMiner*. Barley grain and straw yield were used as supplementary variables while macro- and micronutrients concentration were used as active variables. 

Finally, structural equational modeling (SEM) was used to determine the direct impact of N-source type on macro- and micronutrient content and on barley productivity, which was assessed as latent variable consisted by grain and straw yield. The indirect effect of N-source type on barley productivity through macro- and micronutrient concentration was examined. Due to sample size restrictions, data from all the three growing seasons were merged [29,30]. SEM analysis was performed using *lavaan* package using covariance matrix of the data.

## 3. Results

### 3.1. Seasonal and Nitrogen Fertilization Type Influence Barley Productivity

Both the growing season and the type of nutrient source significantly affected barley yield (F*_6,96_* = 13.57, *p* < 0.01). The grain yield harvested from plots with no N application ranged from 0.91 to 2.3 t/ha during the growing season and was always lower than the harvested yield from plots receiving N fertilization (organic or chemical form). Only, during the first growing season, the addition of compost did not result in an increase in barley grain yield compared to the control. 

**Figure 1:**
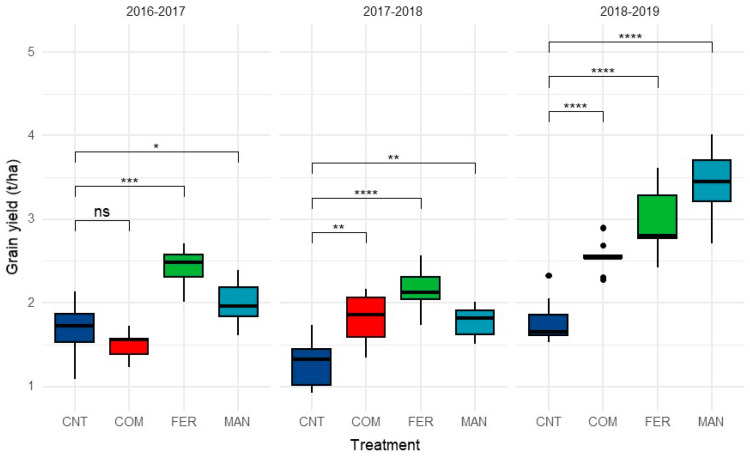
Box plots grain yield of barley grown under different N-source type fertilization schemes for three consecutive growing seasons. In the boxes, median values are indicated. Asterix’s denotes statistically significant differences between the means using Bonferroni adjusted *t*-test (* *p* < 0.05, ** *p* < 0.01, *** *p* < 0.001, **** *p* < 0.0001, ns: no significance).

On the contrary, during the subsequent growing seasons, the addition of any type of input (organic or inorganic) resulted in a substantial increase in barley grain production. The highest yield was noticed in plots that received ammonium nitrate (FER), followed by manure (MAN) at least for the first two growing seasons (Figure 1). Barley grain production was higher in plots treated with goat manure only during the third growing season. Interestingly, a seasonal increasing trend of barley grain yield was observed in plots that received compost (COM), where yield increased from 1.51 t/ha to 2.57 t/ha throughout the growing seasons. (Figure 1). The application of the different N-source had no effect on soil total N, available K, and P-Olsen at the beginning of each growing season and before the implementation of N-source (Supplementary Table S1).

**Figure 2:**
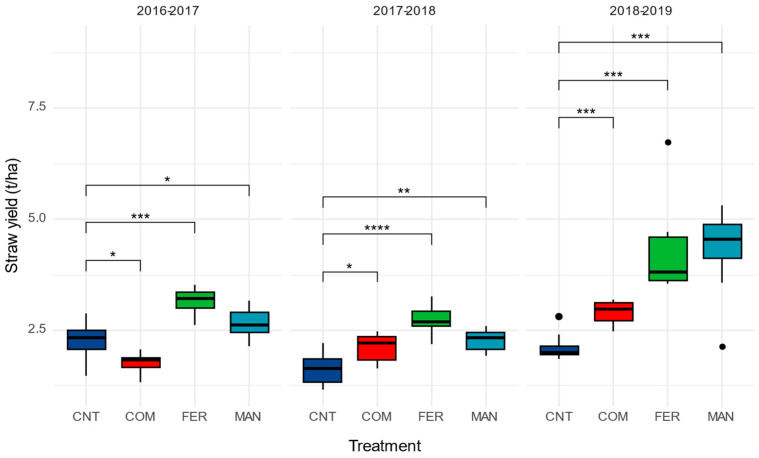
Box plots straw yield of barley grown under different N-source type fertilization schemes for three consecutive growing seasons. Solid lines in the boxes indicate median values. Asterix’s denotes statistically significant differences between the means using Bonferroni adjusted *t*-test (* *p* < 0.05, ** *p* < 0.01, *** *p* < 0.001, **** *p* < 0.0001).

Similar findings were observed in barley straw production, and a significant interaction between N—source type and season was noticed (F*_6,96_* = 4.26, *p* < 0.01). Contrary to the barley grain yield, the application of compost caused a reduction in straw productivity compared to the plots which received no nitrogen input (Figure 2). In 2017–2018 and 2018–2019, the barley straw productivity was higher in plots that received goat manure and fertilizer compared to those of the control treatment (Figure 2).

### 3.2. Seasonal and Nitrogen Fertilization Type Influence Barley Grain Macro-Nutrient Content

Growing season and nitrogen management strategy significantly affected barley grain content in total N, Ca, and Mg. Table 1 depicts the levels of the different nutrients determined as well as the two-way ANOVA results during the different growing seasons, where a significant interaction between the nitrogen fertilization type and the growing season was noticed. The most profound effect of N fertilization, organic or inorganic, on barley grain N content was observed the first growing season (2016–2017), during which N content was substantially increased. Overall, the highest level of N was observed in plots which received ammonium nitrate and goat manure, while barley plants grown in plots with no input had the lower N content. However, this pattern was also affected by the growing season. For example, the total N in barley grains was always higher in plants treated with ammonium nitrate compared to control in all growing seasons. On the contrary, the total nitrogen measured in barley grains which received organic N inputs was different across the growing seasons. For example, barley grains harvested from the compost treated plots exhibited N content similar with that of control. The same pattern was noticed during the last growing season in manure-treated plots.

**Figure 3:**
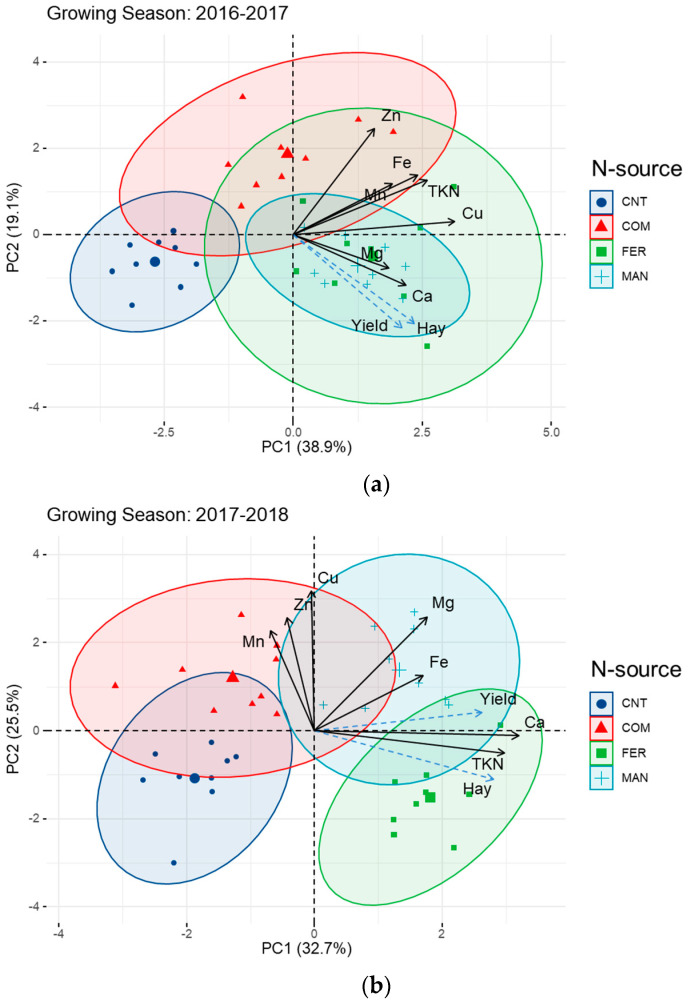

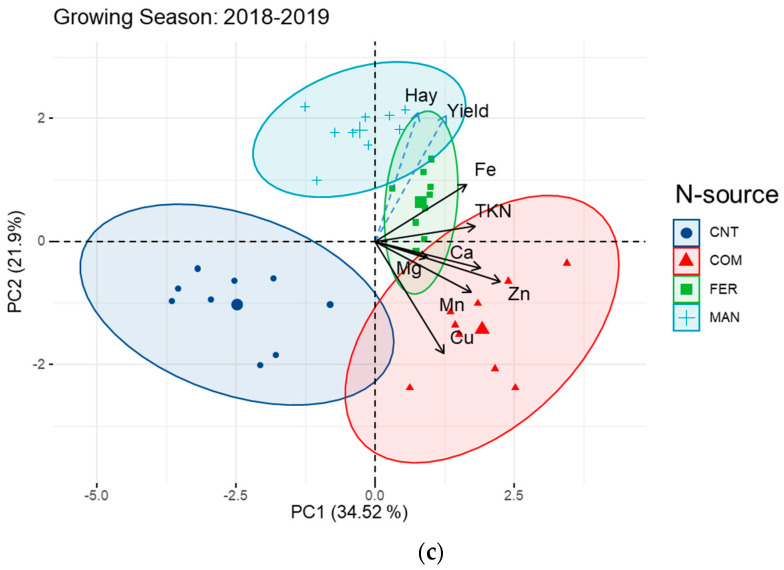
Principal component analysis and biplots of macro- and micronutrient content of barley grains grown under different N-source in three consecutive growing seasons (**a**) 2016–2017, (**b**) 2017–2018 and (**c**) 2019–2020. Solid black lines indicate active variables (macro- and micronutrient content) while dashed light blue lines refer to the dependent variables of straw and grain yield.

The level of Ca in plots receiving organic amendments ranged from 336.5 to 583.1 μg/g dw, with the lowest values to be observed in plots treated with compost during the second growing season (2017–2018). The highest Ca content in barley grain was observed in plots receiving manure (562.7 μg/g dw) during the first growing season (2016–2017) and the second growing season (2017–2018), with 563 and 583 ppm, respectively (Figure 3). During the third growing season (2018–2019), the levels of Ca in barley grain harvested from compost-treated plots was substantially higher (500 ppm) compared to that of manure (418 ppm). The levels of Mg were not consistent, and no clear trend was observed regarding the type of N applied in the different growing seasons, ranging from 669.2 to 904.0 μg/g dw. For example, compost application had no effect on Mg grain content compared with that found in control samples. On the contrary, the application of goat manure and NH_4_NO_3_ caused a significant increase in grain Mg content only during the last growing season, respectively, compared to the control plots (Table 1).

Similarly, the type of N-source significantly affected barley micronutrient content during the different growing seasons. For example, the concentration of Zn was significantly higher in barley grains harvested from compost-treated plots in all growing seasons. Particularly, within the organic N-treated plots (compost vs. manure), the mean amount of Zn measured in the barley grain from the compost plots was 37.3, 41.4, and 46.7 (mg/kg) compared to 29.4, 29.1, and 27.1 (mg/kg) from the manure-treated plots during the three growing seasons, respectively (Table 1). The amount of Mn measured in all growing seasons and treatments ranged from 15.4 to 27.5 (mg/kg). Compost application increased the grain Mn content 1.2 and 2.1 times compared to plants receiving no N fertilization during the first and the second growing season. Interestingly, in these plots, the concentration of Mn measured in barley grains increased constantly over time contrary to the other treatments (Table 1). A strong interaction between season and nitrogen fertilization type was noticed for Cu concentration that ranged from 9.6 to 17.3 mg/kg in all treatments and growing seasons. In control plots, Cu concentration was similar across the different growing seasons. The addition of manure caused a substantial reduction in Cu during the last growing season contrary to the compost plots that exhibited an increased grain Cu content.

### 3.3. Barley Productivity Is Associated with Grain Nutrient Content Based on Nutrient Management within the Different Growing Seasons

The principle component analysis demonstrated the impact of the different type of Nitrogen source during the three growing seasons (Figure 3a–c). The two first principal components explained the 58.0%, 58.2%, and 56.4% of all the parameters examined during the three growing seasons, respectively. Overall, in all growing seasons, higher grain and straw yield were associated with either manure or fertilizer treatments. PCA-biplots clearly showed the preferential association of grain macro- and micro-nutrient content with specific N sources in all growing seasons. For example, the application of compost was associated with an increase in Mn, Zn, and Cu during the second and the last growing season (Figure 3a–c), further supporting the univariate analysis of variance for these nutrients. The concentration of Mn, Zn, and Cu during the second growing season were associated with PC2. During the last growing season, Mn and Zn were associated with PC1 while Cu was associated with PC2. The barley grain and straw yield were positively associated with N, Ca, and Mg content throughout the course of the measurements. These traits were positively related with specific nutrients and this association was seasonal-dependent, further supporting the univariate analysis of variance. For example, the PCA bi-plots and Pearson correlation analysis showed that total N (r = 0.58, *p* < 0.01) and Ca (r = 0.58, *p* < 0.01) content in barley grain were positively correlated with grain yield during the second growing season only, suggesting a strong environmental effect on the assimilation and accumulation of these nutrients in barley grain.

**Figure 4:**
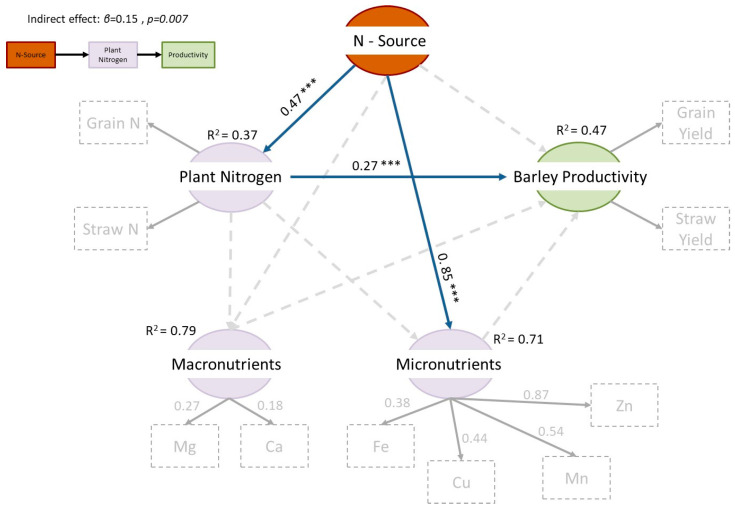
Structural equational modeling analysis results regarding the direct and indirect effects of N-source type on barley productivity, macro- and micronutrient content. Explained variance of the latent variables is presented next to each variable. Numbers next to path arrows are the standardized coefficients to account the effect sizes for significant associations (*p* < 0.05). Faint and dashed path arrows are not significant while blue path arrows denote statistically significant associations. Model fit was estimated using χ^2^, *p*-value (<0.05), comparative fit index (CFI > 0.9), root mean square error of approximation (0.06 ≤ RMSEA ≤ 0.08), standardized root-mean-square of the residuals (SRMR ≤ 0.08), and Akaike information criteria (AIC). Full results of the model analysis are presented in the Supplementary data section. *** *p* < 0.001.

The structural equational modeling analysis results regarding the direct and indirect effects of N-source type on barley productivity, macro- and micronutrient content are depicted in Figure 4 for all the three growing seasons. The developed model was significant (*p* < 0.05) and exhibited acceptable fitting criteria (χ^2^ =61.35, *p* = 0.001, CFI= 0.92, RMSEA = 0.08, SRMR = 0.07, AIC = 2591.4). The full analysis is presented in Supplementary Data. The results revealed that nitrogen source was positively associated with barley N (0.47, *p* < 0.001) and micronutrient content (0.85, *p* < 0.001) but not with macronutrients (0.71, *p* = 0.243). Barley productivity was associated directly only with N content of the plant (0.27, *p* = 0.002) and indirectly from the N source used again through the accumulation of N in both grain and straw (β = 0.15, *p* = 0.007) (Figure 4).

## 4. Discussion

Under the semi-arid conditions of Cyprus and the Eastern Mediterranean region, the climate variability and the N-inputs are affecting barley gain and straw yield as well as grain quality in terms of macro- and micronutrient content. A recent meta-analysis report showed that in the Mediterranean basin, the response of barley to the nitrogen addition was remarkable and led to a substantial increase in barley productivity, especially in agro-ecosystems with low productivity [31]. Previously, it was shown that sustainable nitrogen management strategies could alleviate the negative effects of water shortage and improve cereal performance in marginal arid environments [32]. The response of barley to nitrogen addition has been attributed to an increase in the water use efficiency of the crop under rainfed conditions [33], while the inclusion of organic amendments could further enhance the crop’s water uptake [34]. In line with these reports, in the current study, both the organic and chemical N fertilization caused a significant increase in barley grain and straw production (Figure 1 and Figure 2). Interestingly, the progressive increase in grain and straw yield in compost-treated plots suggests that there is a beneficial residual effect of compost to the crop. Indeed, during the first growing season, in the compost-treated plots, the grain yield was similar to that found in the control, while the straw yield was significantly lower (Figure 1 and Figure 2). These results suggest that the application of compost could suppress the barley productivity. This effect could be possibly due to changes on soil nutrient availability. As an example, composts with high C/N ratio could stimulate microbial activity and immobilize nitrogen [35], which, in turn, could affect crop productivity. Indeed, the C/N ratio of the compost applied was 24.6, which supports slower N release that would affect N availability during the critical developmental stages of the crop. Pairwise comparisons showed that barley plants receiving the compost exhibited lower grain and straw yield compared with that receiving goat manure. This further supports that nutrient availability could be the main reason for this reduction. Previous studies demonstrated that the composts, especially those derived from green residues, are poor in nutrients and their importance for plant growth is related to their beneficial effect on soil physical properties in the long term [36,37]. Apparently, the positive response of barley to the compost addition could be related to an overall improvement of soil fertility through the replenishment of soil carbon stocks and the increase in soil microbial activity, functioning and biodiversity, [38,39] while under some conditions, composts could provide pathogen suppression [40] in the long term.

The barley grain mineral composition greatly influences its nutritional quality and it is important for human and animal nutrition. This study showed that N-source type affected the accumulation of the different macro- and micronutrients; however, this was dependent on the growing season. The uptake of macro- and micronutrients is affected by the interactions between the different available nutrients in the soil, and the nutrients externally applied. In addition, the differences in the nutrient management practices are affecting soil functioning and plant metabolism, while they are also shaping the microbial community structure, which in turn affects nutritional quality and plant composition [41]. Previous studies showed that micronutrient content increases after fertilization [42]. For example, nitrogen and phosphorus management has been identified as significant components controlling Zn concentration in cereals. The application of optimum N rates increases the Zn accumulation in wheat grain [43]. In the current study, the micronutrient content in barley grain was highly and significantly associated with N-source (Figure 4) and particularly with the application of organic amendments. For example, the repeated application of compost resulted in increased Zn concentration in barley grain over time, suggesting a more efficient utilization of this micronutrient from the soil. Similarly, Aghili et al. [44] showed that the incorporation of green manure caused a substantial increase in grain Zn concentration in bread wheat. The higher concentration of Zn found in the compost-treated plots could also be related to an increase in the bioavailability of this micronutrient. It is well established that the addition of organic amendments increases the amount of soil-organic carbon, which is significantly correlated with the increase in soil Zn availability [45]. The mechanisms responsible for the increase in Zn availability are not yet known, however, and further research is required.

## 5. Conclusions

The current study demonstrated that the productivity of rainfed barley ecosystems differs between the growing season, but type of the N-source is a main factor determining the productivity of the crop within each growing season. These findings further support the notion that nitrogen fertilization under arid conditions could be beneficial for barley cropping. The effect of N-source type on barley productivity is indirect and is associated with the nitrogen accumulation in grain and straw. Macro- and micronutrients are not directly associated with barley performance, but the micronutrients content in barley grain content is highly and significantly associated with the N-source type. Micronutrients have been particularly enriched in plants receiving organic amendments. Further studies are needed to reveal the mechanisms responsible for the observed enhanced accumulation of micronutrients in barley grain when organic amendments are applied under arid or semi-arid conditions.

## Figures and Tables

**Table 1:** Concentration of macro- and micronutrients in barley grain harvested during the three consecutive growing seasons under the different N-source type applications. The source of variance was calculated with two-way ANOVA. Different letters denote statistically significant differences between the mean concentration within each growing season based on TukeyHSD at *p* < 0.05.

Growing Season	Nitrogen Management	Total N	Ca	Mg	Zn	Mn	Cu	Fe
		(%)	(μg/g dry weight)					
2016–2017	Control	1.3 ± 0.3 a	400.5 ± 29.2 a	711.4 ± 75.5 a	17.7 ± 1.9 a	16.8 ± 1.5 a	9.6 ± 2.1 a	41 ± 8.6 a
	Compost	2.3 ± 1.1 b	420.0 ± 62.2 ab	759.2 ± 56.8 a	37.3 ± 5.2 c	18.6 ± 3.4 ab	13.6 ± 1.5 b	53.6 ± 2.1 b
	NH_4_NO_3_	2.4 ± 0.5 b	466.2 ± 35.7 b	768 ± 113.9 ab	26.3 ± 5.0 b	19.7 ± 1.9 b	16.4 ± 2.1 c	53.6 ± 7.2 b
	Manure	2.3 ± 0.1 b	562.7 ± 74.5 c	896.6 ± 86.5 b	29.4 ± 1.9 b	17.7 ± 0.6 b	14.2 ± 1.9 bc	50.7 ± 5.6 ab
2017–2018	Control	1.4 ± 0.4 a	383.0 ± 47.1 b	678.9 ± 69.8 a	18.7 ± 3.9 a	19.0 ± 1.7 a	10.5 ± 2.6 ab	51.4 ± 8.9 a
	Compost	1.8 ± 0.3 a	336.5 ± 18.6 a	757.9 ± 89 ab	41.4 ± 4.0 c	22.8 ± 2.6 b	12.7 ± 1.2 bc	49 ± 4.8 a
	NH_4_NO_3_	2.7 ± 0.2 b	555.5 ± 43.7 c	716.3 ± 99.6 a	22.3 ± 9.4 ab	18.9 ± 2.2 a	9.3 ± 0.4 a	51.7 ± 2.7 a
	Manure	2.2 ± 0.4 b	583.1 ± 34.4 c	875.7 ± 118.1 b	29.1 ± 3.2 b	20.8 ± 4.6 ab	14 ± 1.4 c	66.6 ± 3.3 b
2018–2019	Control	1.7 ± 0.6 a	354.0 ± 87.3 a	724.1 ± 140.8 ab	16 ± 11.4 a	15.4 ± 5.0 a	10.7 ± 1.8 b	42.1 ± 10.9 a
	Compost	2.5 ± 0.5 ab	499.9 ± 44.3 b	783.4 ± 156.6 ab	46.7 ± 2.0 c	27.5 ± 6.3 b	17.3 ± 3.1 d	53.5 ± 11.4 a
	NH_4_NO_3_	2.5 ± 0.2 b	457.1 ± 39.8 b	904 ± 170.8 b	27.9 ± 3.4 b	18.5 ± 1.0 a	14.1 ± 2.1 c	53.2 ± 2.6 a
	Manure	2.1 ± 0.7 ab	418.1 ± 93.4 a	669.2 ± 74.4 a	27.1 ± 1.9 b	18.4 ± 2.9 a	5.9 ± 2.7 a	52.1 ± 3.3 a
		Source of Variance						
Growing season		ns	*	*	*	*	*	*
N-management		*	*	*	*	*	*	*
Interaction		*	*	*	*	*	*	*

ns: no significance; * *p* < 0.05.

## Data Availability

The data presented in this study are available on request from the corresponding author.

## Supplementary Materials

The following supporting information can be downloaded at: https://www.mdpi.com/article/10.3390/ijerph20053943/s1.
