# Supplementary material for: N-Source Determines Barley Productivity, Nutrient Accumulation, and Grain Quality in Cyprus Rainfed Agricultural Systems
Source: Int J Environ Res Public Health. 2023 Feb 23;20(5):3943. doi: 10.3390/ijerph20053943 (PMC10001598; doi:10.3390/ijerph20053943)
Supplement: Supplementary file 1 [file ijerph-20-03943-s001.zip › ijerph-2188079-supplementary.pdf]

**SUPPLEMENTARY DATA**

**Table 1:** Concentration of soil total N, available K and P-Olsen at the beginning of each growing season and before the application of the treatments and seeding the crop. The source of variance was calculated with two-way ANOVA. Different letters denote statistically significant differences between the mean concentration within each growing season based on TukeyHSD at $p<0.05$

| Growing season | Nitrogen management | Total N (mg/g) | K (ppm) | P-Olsen (ppm) |
|---|---|---|---|---|
| 2016-2017 | Control | 0.91±0.12 a | 200.1±11.8 a | 25.5±4.9 a |
| | Compost | 1.12±0.14 a | 215.4±12.3 a | 27.3±3.8 a |
| | $NH_4NO_3$ | 0.98 ±0.11 a | 210.5±15.4 a | 25.6±5.4 a |
| | Manure | 0.92±0.09 a | 221.8±9.6 a | 26.4±3.3 a |
| 2017-2018 | Control | 0.89±0.09 a | 210.3±12.7 a | 20.7±5.4 a |
| | Compost | 0.98±0.11 a | 205.6±22.7 a | 34.2±7.8 a |
| | $NH_4NO_3$ | 0.93±0.09 a | 199.1±38.5 a | 22.6±4.6 a |
| | Manure | 1.21±0.12 a | 208.4±23.7 a | 31.3±6.9 a |
| 2017-2018 | Control | 0.92±0.09 a | 214.6±23.7 a | 19.5±5.3 a |
| | Compost | 1.42±0.11 a | 235.7±35.1 a | 29.4±4.8 a |
| | $NH_4NO_3$ | 1.12±0.13 a | 220.9±18.3 a | 24.2±3.6 a |
| | Manure | 1.26±0.14 a | 245.6±26.4 a | 27.9±2.8 a |
| | | Source of Variance | | |
| Growing season | | ns | ns | ns |
| N-management | | ns | ns | ns |
| Interaction | | ns | ns | ns |

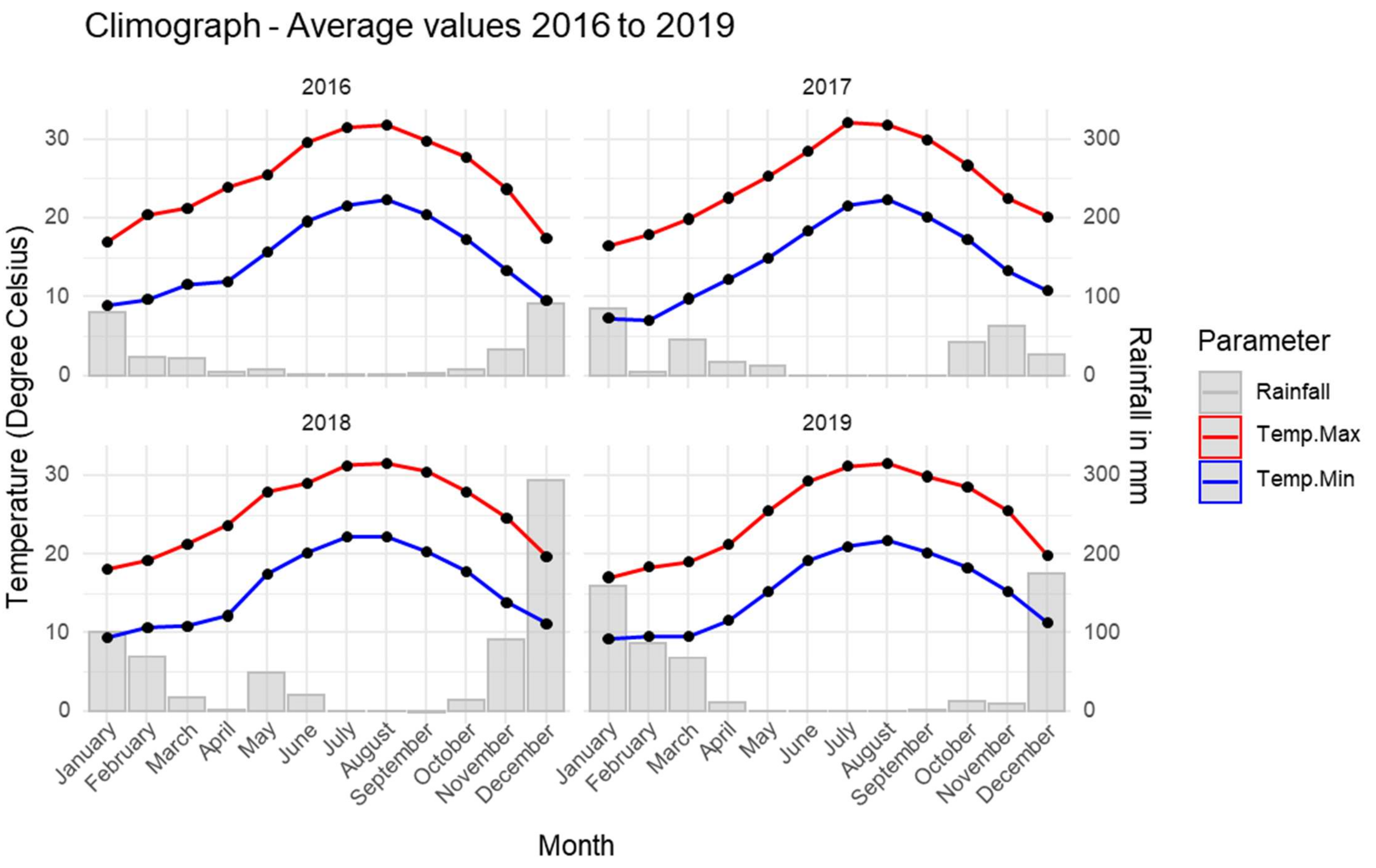

Climograph - Average values 2016 to 2019
2016
2017
2018
2019
Temperature (Degree Celsius)
Rainfall in mm
Month
0
10
20
30
100
200
300
January
February
March
April
May
June
July
August
September
October
November
December
Parameter
Rainfall
Temp.Max
Temp.Min

lavaan 0.6-12 ended normally after 89 iterations

| | |
|---|---|
| Estimator | ML |
| Optimization method | NLMINB |
| Number of model parameters | 24 |
| Number of observations | 108 |

Model Test User Model:

| | |
|---|---|
| Test statistic | 61.352 |
| Degrees of freedom | 30 |
| P-value (Chi-square) | 0.001 |

Parameter Estimates:

| | |
|---|---|
| Standard errors | Standard |
| Information | Expected |
| Information saturated (h1) model | Structured |

Latent Variables:

| | Estimate | Std.Err | z-value | P(>\|z\|) | Std.lv | Std.all |
|---|---|---|---|---|---|---|
| Nitrogen =~ | | | | | | |
| TKN_GR (l1) | 1.000 | | | | 0.995 | 1.000 |
| TKN_HAY (l2) | 0.455 | 0.177 | 2.569 | 0.010 | 0.343 | 0.345 |
| macro =~ | | | | | | |
| Ca (l3) | 1.000 | | | | 0.146 | 0.177 |
| Mg (l4) | 2.336 | 2.121 | 1.101 | 0.271 | 0.342 | 0.274 |
| micro =~ | | | | | | |
| Cu (l5) | 1.000 | | | | 0.432 | 0.443 |
| Zn (l6) | 2.028 | 0.462 | 4.388 | 0.000 | 0.877 | 0.871 |
| Mn (l7) | 1.245 | 0.335 | 3.720 | 0.000 | 0.538 | 0.541 |
| Fe (l8) | 0.854 | 0.287 | 2.975 | 0.003 | 0.369 | 0.379 |
| Prod =~ | | | | | | |
| Yield (l9) | 1.000 | | | | 0.920 | 0.926 |
| Straw (l10) | 1.058 | 0.109 | 9.689 | 0.000 | 0.973 | 0.980 |

Regressions:

| | Estimate | Std.Err | z-value | P(>\|z\|) | Std.lv | Std.all |
|---|---|---|---|---|---|---|
| Nitrogen ~ | | | | | | |
| Nsrc (a1) | 0.470 | 0.085 | 5.532 | 0.000 | 0.472 | 0.470 |
| macro ~ | | | | | | |
| Nsrc (a2) | 0.104 | 0.090 | 1.167 | 0.243 | 0.714 | 0.710 |
| micro ~ | | | | | | |
| Nsrc (a3) | 0.363 | 0.083 | 4.366 | 0.000 | 0.840 | 0.836 |
| Prod ~ | | | | | | |
| Nsrc (a4) | 0.032 | 1.392 | 0.023 | 0.981 | 0.035 | 0.035 |
| micro (a6) | -0.854 | 0.529 | -1.613 | 0.107 | -0.401 | -0.401 |
| macro (a7) | 3.485 | 13.023 | 0.268 | 0.789 | 0.555 | 0.555 |
| Nitrogen (a8) | 0.298 | 0.096 | 3.095 | 0.002 | 0.322 | 0.322 |

Variances:

| | Estimate | Std.Err | z-value | P(>\|z\|) | Std.lv | Std.all |
|---|---|---|---|---|---|---|
| .TKN_GR | 0.000 | | | | 0.000 | 0.000 |
| .Ca | 0.969 | 0.138 | 7.034 | 0.000 | 0.969 | 0.978 |
| .Mg | 0.874 | 0.248 | 3.522 | 0.000 | 0.874 | 0.882 |
| .Cu | 0.804 | 0.113 | 7.092 | 0.000 | 0.804 | 0.811 |
| .Zn | 0.222 | 0.078 | 2.831 | 0.005 | 0.222 | 0.224 |
| .Mn | 0.701 | 0.102 | 6.885 | 0.000 | 0.701 | 0.708 |
| .Fe | 0.854 | 0.119 | 7.173 | 0.000 | 0.854 | 0.862 |
| .Yield | 0.140 | 0.082 | 1.720 | 0.085 | 0.140 | 0.142 |
| .Straw | 0.038 | 0.089 | 0.427 | 0.669 | 0.038 | 0.039 |
| .Nitrogen | 0.772 | 0.105 | 7.348 | 0.000 | 0.779 | 0.779 |
| .macro | 0.011 | 0.041 | 0.261 | 0.794 | 0.495 | 0.495 |
| .micro | 0.056 | 0.028 | 1.992 | 0.046 | 0.301 | 0.301 |
| .Prod | 0.557 | 0.520 | 1.069 | 0.285 | 0.658 | 0.658 |

R-Square:

| | Estimate |
|---|---|
| TKN_GR | 1.000 |
| Ca | 0.022 |
| Mg | 0.118 |
| Cu | 0.189 |
| Zn | 0.776 |
| Mn | 0.292 |
| Fe | 0.138 |
| Yield | 0.858 |
| Straw | 0.961 |
| Nitrogen | 0.371 |
| macro | 0.792 |
| micro | 0.711 |
| Prod | 0.472 |

Defined Parameters:

| | Estimate | Std.Err | z-value | P(>\|z\|) | Std.lv | Std.all |
|---|---|---|---|---|---|---|
| nitrogen_a1a8 | 0.140 | 0.052 | 2.701 | 0.007 | 0.152 | 0.152 |
| macro_a2a7 | 0.364 | 1.378 | 0.264 | 0.792 | 0.396 | 0.394 |
| micro_a3a6 | -0.310 | 0.187 | -1.656 | 0.098 | -0.337 | -0.336 |
